# Nutritional Status and Falls in Community-Dwelling Older People: A Longitudinal Study of a Population-Based Random Sample

**DOI:** 10.1371/journal.pone.0091044

**Published:** 2014-03-10

**Authors:** Ming-Hung Chien, How-Ran Guo

**Affiliations:** 1 Department of Health Care Administration, Chung Hwa University of Medical Technology, Tainan, Taiwan; 2 Department of Environmental and Occupational Health, College of Medicine, National Cheng Kung University, Tainan, Taiwan; 3 Department of Occupational and Environmental Medicine, National Cheng Kung University Hospital, Tainan, Taiwan; University of Southampton, United Kingdom

## Abstract

**Background:**

Falls are common in older people and may lead to functional decline, disability, and death. Many risk factors have been identified, but studies evaluating effects of nutritional status are limited. To determine whether nutritional status is a predictor of falls in older people living in the community, we analyzed data collected through the Survey of Health and Living Status of the Elderly in Taiwan (SHLSET).

**Methods:**

SHLSET include a series of interview surveys conducted by the government on a random sample of people living in community dwellings in the nation. We included participants who received nutritional status assessment using the Mini Nutritional Assessment Taiwan Version 2 (MNA-T2) in the 1999 survey when they were 53 years or older and followed up on the cumulative incidence of falls in the one-year period before the interview in the 2003 survey.

**Results:**

At the beginning of follow-up, the 4440 participants had a mean age of 69.5 (standard deviation  = 9.1) years, and 467 participants were “not well-nourished,” which was defined as having an MNA-T2 score of 23 or less. In the one-year study period, 659 participants reported having at least one fall. After adjusting for other risk factors, we found the associated odds ratio for falls was 1.73 (95% confidence interval, 1.23, 2.42) for “not well-nourished,” 1.57 (1.30, 1.90) for female gender, 1.03 (1.02, 1.04) for one-year older, 1.55 (1.22, 1.98) for history of falls, 1.34 (1.05, 1.72) for hospital stay during the past 12 months, 1.66 (1.07, 2.58) for difficulties in activities of daily living, and 1.53 (1.23, 1.91) for difficulties in instrumental activities of daily living.

**Conclusion:**

Nutritional status is an independent predictor of falls in older people living in the community. Further studies are warranted to identify nutritional interventions that can help prevent falls in the elderly.

## Introduction

Falls are one of the most serious problems among the older people. Approximately 30% of community-dwelling people 65 years and older have at least one fall each year [1], [2]. In Chinese, 14.7% to 34% of older people experience falls annually [3]–[5], and 60% to 75% of them may suffer from serious injuries such as fractures (6% to 8%) [4]. Falls and fall injuries can lead to functional decline, disability, and death in older people [6], [7]. In the United States, 15802 persons 65 years or older died from falls related injuries in 2005 [8].

A number of studies have been conducted to determine the risk factors associated with falls in older people. The identified risk factors for falls include demographic characteristics (such as older age [3]–[5], [9], [10], female gender [4], [5], [10], [11], unmarried status [10], [12], and low educational level [10]), anthropometric measurements (such as larger waist circumference [5]), health status (such as use of medication [4], [13]–[15], low physical health [2], [9], [10], [16], cognitive impairment [14], [17], mobility impairment [1], [13]–[15], [17], [18], visual impairment [19], history of falls [1], [3], [20], [21], depression [17], [21], and malnutrition [22]–[25]), and health behavior (such as decline in activities of daily living [4], [11], low or high mobility level [9], [10], [13], and alcohol consumption [16], [26]).

Malnutrition is common in older people and associated with impaired muscle function, reduced cognitive function, decreased bone mass, and increased morbidity [27], [28]. The Mini Nutritional Assessment (MNA) was developed for measuring geriatric nutritional status [28]–[35], and some studies have shown that adoption of population-specific cut-offs for body mass index (BMI), calf circumference (CC), and mid-arm circumference (MAC) could improve the prediction of MNA for nutritional status in older Taiwanese people [36]–[40].

Some studies have shown that malnutrition was associated with falls or able to predict falls of older people [22]–[25]. However, most of them were conducted in Western populations who were hospitalized or in home care or long-term care. Using the key words “fall” combing with “older people,” “elderly,” and “aged” to search literature in the PubMed and Chinese Electronic Periodical Services up to the end of December 2012, we failed to find any studies evaluating nutritional status as a predictor for falls in older people living in community dwellings using a population-based sample. The objective of this longitudinal study is to determine whether nutritional status is a predictor of falls in older people living in the community, which is a much larger population than those who were institutionalized or in home care, and thereby to obtain results that will have a broader application.

## Methods

### Participants

The Survey of Health and Living Status of the Elderly in Taiwan (SHLSET) is a longitudinal population-based cohort survey carried out by the Bureau of Health Promotion (BHP) of the Department of Health. The survey adopts a multistage sampling frame. The first stage involves the selection of 56 primary sampling units (PSUs) from the 331 non-aboriginal townships in Taiwan through systematic random sampling. In the second stage, blocks of the smallest administrative division in Taiwan (called “lin”) are randomly selected in each PSU chosen from the first stage. In the third stage, two eligible respondents are selected by systematic random sampling from each block (lin) chosen from the second stage. Because a random sample is drawn in each stage, the final sample is a random sample representing the national population aged 60 years or older. Since its initiation in 1989, the survey has been conducting periodic interviews, and participants receive individual, face-to-face, in-home interviews by well-trained interviewers [41].

In the third survey conducted in 1996, new members 50–66 years of age were selected through the same method and added to the sample, extending the cohort to members as young as 50 years old. In the fourth survey in 1999, when the original cohort members were 70 years or older and the 1996 cohort members were 53–69 years old, an MNA was added to the questionnaire to evaluate the nutritional status of the participants. The numbers of respondents and response rates in each survey are shown in Table 1.

**Table:** **Table1.** Numbers of respondents and response rates.

Survey Year	Age at Sampling	Respondents	Response rate	Population
1989	≥60 years old	4049	91.8%	1724950
1996	≥67 years old	2669	88.9%	1315664
	50–66 years old	2462	81.2%	2657461

We adopted participants of the 1999 survey as our study population and followed them up using data collected in the fifth survey in 2003. After excluding participants with incomplete data, we included 1743 participants (91.0% of the original cohort members who were still alive) 74 years or older and 2035 participants (92.1% of the 1996 new cohort members who were alive) 57–73 years old in our study population. (Table 1)

### Assessment of Nutritional Status

The MNA used in the 1999 SHLSET included 18 items assessing four domains: anthropometric measurements (weight loss, BMI, MAC, and CC), a general assessment (lifestyle, medication, mobility, and presence of signs of depression or dementia), a dietary assessment (number of meals, food and fluid intake, and autonomy of feeding), and a subjective assessment (self perception of health and nutrition). A small modification was made to Item B, and the question became “Did you lose more than 3 kg of weight during last year?” To account for cultural and anthropometric differences across populations, nutritional assessment tools should be as much population- or race-specific as possible [36], and a population-specific MNA was found to be capable of effectively predicting the nutritional status of Taiwanese people [39], [42]. Therefore, we adopted the MNA Taiwan Version 2 (MNA-T2), which replaced BMI by population-specific CC in the scale, used population-specific MAC and CC cut-offs instead of values specified in the original MNA [37], [39], and reassigned new scores to MAC and CC in order to keep the full score as 30 points (Table 2). MAC and CC were thought to be better indicators of nutritional status and health conditions than BMI for older people [43], [44], and it has been demonstrated that adoption of MNA-T2 has improved the prediction in older Taiwanese people [38]–[40], [45]. In the current study, the nutritional status of participants was categorized into three groups and defined according to the MNA-T2 scores: “well-nourished” (≥24 points), “at risk of malnutrition” (17.0–23.5 points), and “malnutrition” (<17.0 points) [28], [46], [47], and we compared those who were “well-nourished” (>23.5 points) to those who were “not well-nourished” (≤23.5 points).

**Table 2:** Distribution of the scores of MNA-T2^a^ in the participants in 1999.

Items	Score	Number (%)
A. Food intake declined over the past 3 month		
Severe	0	379 (8.5)
Moderate	1	12 (0.3)
No decline	2	4049 (91.2)
B. Weight loss >3 kg during last year		
Yes	0	636 (14.3)
Does not know	1	25 (0.6)
No	3	3779 (85.1)
C. Mobility		
Bed or chair-bound	0	107 (2.4)
Able to get out bed but does not go out	1	181 (4.1)
Goes out	2	4152 (93.5)
D. Psychological stress/acute disease during past 3 months		
Yes	0	411 (9.3)
No	2	4029 (90.7)
E. Neuropsychological problems^b^		
Severe dementia or depression	0	4 (0.1)
Mild dementia	1	27 (0.6)
No psychological problems	2	4347 (97.9)
R. Calf circumference (cm)		
<28 in men, <25 in women	0	101 (2.3)
28–29 in men, 25–26 in women	1	72 (1.6)
>29–30 in men, >26–27 in women	2	97 (2.2)
>30 in men, >27 in women	3	4170 (93.9)
G. Live independently^c^		
No	0	61 (1.4)
Yes	1	4379 (98.6)
H. Takes 4 or more kinds of prescribed drugs/day^d^		
Yes	0	152 (3.4)
No	1	4288 (96.6)
J. Full meals daily		
1	0	4 (0.1)
2	1	187 (4.2)
3	2	4249 (95.7)
K. Daily consumption of protein-rich food^e^		
0 or 1 “yes”	0	87 (2.0)
2 “yes”	0.5	485 (10.9)
3 “yes”	1	3868 (87.1)
L. Consumes ≥2 servings of fruits or vegetables per day		
No	0	323 (7.3)
Yes	1	4117 (92.7)
N. Mode of feeding		
Unable to eat without assistance	0	75 (1.7)
Self-fed with difficulty	1	99 (2.2)
Self-fed without any problem	2	4266 (96.1)
O. Self view of nutritional status		
Views self as being malnourished	0	338 (7.6)
Uncertain of nutritional status	1	435 (9.8)
No nutritional problem	2	3667 (82.6)
P. Self view of health status compared to peers		
Not as good	0	1869 (42.1)
As good	1	2346 (52.8)
Better	2	225 (5.1)
Q. Mid-arm circumference (cm)		
<22.5 in men, <21.0 in women	0	111 (2.5)
22.5–23.5 in men, 21.0–22.0 in women	1	109 (2.5)
>23.5 in men, >22.0 in women	2	4220 (95.0)
MNA-T2^f^		
≤16.5 (malnutrition)		58 (1.3)
17.0–23.5 (at risk of malnutrition)		409 (9.2)
≥24.0 (well-nourished)		3911 (88.1)

a MNA-T2: Modified Mini Nutritional Assessment Taiwan Version 2.

b Assessed by the number of correct answers to the questions “Where is this place?” and “How old are you?” that were asked by the interviewer.

c Defined as not living in a nursing home or hospital.

d Include both Western and traditional Chinese medications.

e Protein rich foods include meat, poultry, fish, seafood, eggs, dairy products, and legumes.

f Scores could not be calculated for 62 participants (1.4%).

A review of literature has shown that MNA has high reliability, specificity, and sensitivity [46], and MNA has been validated in some Western and non-Western populations for assessing older people at risk for malnutrition [37], [39], [48]. It has been shown to be capable of identifying people at risk for malnutrition before severe changes in weight or serum proteins occur [34], [46] and to be a better indicator in detecting emerging malnutrition than BMI, weight loss, or energy intake [48]. In addition, recent studies in community-living elderly in Taiwan found that MNA-T2 was valid in predicting the nutritional status and long-term mortality of older Taiwanese people and had superior predictive abilities in comparison with the original MNA [43], [45].

### Assessment of Falls

In the 2003 SHLSET, a “fall” was defined as a fall or slip due to any causes whether it caused injuries or not. The participants were asked to recall whether they had any falls in the one-year period before the interview, and therefore data collected in the 2003 survey can be adopted to assess the cumulative incidence in the one-year period.

### Assessment of Other Potential Risk Factors

In addition to nutritional status, we adopted the data collected in the 1999 SHLSET to evaluate the effects of other potential risk factors on falls, including gender, age, marital status, years of formal education, living alone, smoking, regular alcohol consumption, no routine exercise, neuroactive medications use, hypnotics use, sedative use, calcium supplement consumption, assistive devices use, Geriatric Depression Score measured by the short version of the Center for Epidemiologic Study Depression Scale (CES-D), history of falls, hospital stays during past 12 months, visits to the emergency departments of hospitals (ED visits) during past 12 months, difficulties in activities of daily living (ADL), and difficulties in instrumental activities of daily living (IADL). Neuroactive medications use was defined as taking psychoactive and stimulant medications on a daily basis. Hypnotics referred to any pills that help sleeping, and sedative referred to any drugs that were used to calm down the participants. Calcium supplement consumption was defined as taking calcium supplement pills, with or without vitamin D, on a daily basis. Assistive devices use was defined as the use of a cane, crutch, or walker for assisting walking. Difficulties in ADL were defined as any difficulties in bathing, dressing, feeding, transferring (such as getting off the bed, standing up, and sitting down on a chair), walking, or toileting. Difficulties in IADL were defined as any difficulties in shopping, doing laundry, preparing meal, handling finances, handling transportation, doing housework, housekeeping, managing medication, or using telephone.

### Statistical Analyses

We used χ^2^ tests to evaluate differences in the distributions of categorical variables between two groups of participants. Logistic regressions were conducted to indentify the risk factors for falls and evaluate their effects. Although each member in the same cohort carries the same weight, our study population included both members in the original cohort who were ≥70 years old with a sampling probability of 1/493 and members in the 1996 cohort who were 53–69 years old with a sampling probability of 1/1079. Therefore, we applied a weight of 1.4 to participants 53–69 years of age in 1999, and a weight of 0.64 to participants ≥70 years old. In the multivariate regression analyses, we constructed a full model which included all potential risk factors and then, through stepwise logistic regressions, a reduced model in which all the independent variables were statistically significant. We performed all analyses using the SPSS software Version 17.0 (Statistical Package for the Social Sciences, Chicago, IL) at the two-tailed significance level of 0.05.

### Ethics

The SHLSET was conducted by the Department of Health (DOH) of the Taiwan government, which is the governing body of ethics regarding human health research in the nation. Data collected in the SHLSET are publicly available for academic use, but before releasing the data through its Collaboration Center of Health Information Application, DOH needs to review and approve the study proposals. DOH waived the need of a written consent for collecting the data as the personal identifiers were removed from the database released. The purpose of SHLSET is to describe the health and living status of Taiwanese elderly, not the risk factors of falls, and nutritional status was not evaluated in every round of SHLSET. We conducted this study through secondary data analyses by adopting the data collected in the SHLSET and were able to complete the study because the DOH had reviewed and approved the study proposal. This study was conducted according to the principles expressed in the Declaration of Helsinki, and the data were analyzed anonymously.

## Results

### Characteristics of Participants

In 1999, the 4440 participants had a mean age of 69.5 (standard deviation [SD]  = 9.1) years, with 2978 (67.1%) were 65 years or older. There were slightly more men (2358; 53.1%), and 2992 (67.4%) of the participants had spouses (defined as being married or living with a cohabiting companion). A large proportion (77.5%) of the participants had no more than 6 years of formal education. Only 62 (1.4%) of the participants did not have MNA-T2 scores, and all of them were due to the lack of information on neuropsychological problems (Item E in Table 2). We compared them to those who had MNA-T2 scores and found no significant differences in gender, marital status, and years of formal education. However, the participants who had MNA-T2 scores were younger than those who did not (Table 3). In comparison with those who were included in the analysis (n = 3497), those who were included had higher proportions of being a male, with an age between 53 and 74 years old, and having a spouse (Table 4).

**Table 3:** Demographic characteristics of participants with and without MNA-T2^a^ data at baseline.

	Number (%)			P Value
Characteristics	With	Without	Total	
Gender				0.129
Men	2331 (53.2)	27 (43.5)	2358 (53.1)	
Women	2047 (46.8)	35 (56.5)	2082 (46.9)	
Age (year)				<0.001
53–74	3050 (69.7)	27 (43.5)	3077 (69.3)	
75–84	1125 (25.7)	25 (40.3)	1150 (25.9)	
≥85	203 (4.6)	10 (16.1)	213 (4.8)	
Marital status				0.064
Has a spouse^b^	2957 (67.5)	35 (56.5)	2992 (67.4)	
No spouse	1421 (32.5)	27 (43.5)	1448 (32.6)	
Years of formal education				0.866
≤6	3393 (77.5)	50 (80.6)	3443 (77.5)	
7–9	422 (9.6)	5 (8.1)	427 (9.6)	
10–12	311 (7.1)	3 (4.8)	314 (7.1)	
>12	252 (5.8)	4 (6.5)	256 (5.8)	

a MNA-T2: Modified Mini Nutritional Assessment Taiwan Version 2.

b Including those who were married and those who had a cohabiting companion.

**Table 4:** Comparison of the subjects included and not included in the analysis.

	Number (%)			P Value
Characteristics	Included	Not Included	Total	
Gender				<0.001
Men	1805 (51.6)	526 (59.7)	2331 (53.2)	
Women	1692 (48.4)	355 (40.3)	2047 (46.8)	
Age (year)				<0.001
53–74	2622 (75.0)	428 (48.6)	3050 (69.7)	
75–84	782 (22.4)	343 (38.9)	1125 (25.7)	
≥85	93 (2.6)	110 (12.5)	203 (4.6)	
Marital status				<0.001
Has a spouse^a^	2483 (71.0)	474 (53.8)	2957 (67.5)	
No spouse	1014 (29.0)	407 (46.2)	1421 (32.5)	
Years of formal education				0.134
≤6	2687 (76.8)	706 (80.1)	3393 (77.5)	
7–9	342 (9.8)	80 (9.1)	422 (9.6)	
10–12	262 (7.5)	49 (5.6)	311 (7.1)	
>12	206 (5.9)	46 (5.2)	252 (5.8)	

a Including those who were married and those who had a cohabiting companion.

### Nutritional Status

The mean score of the 4378 participants who had data for calculating MNA-T2 at baseline (in 1999) was 26.8 (SD = 2.9), and 467 (10.5%) of them were not well-nourished, including 409 (9.2%) being at risk of malnutrition and 58 (1.3%) being malnourished (Table 2).

### Cumulative Incidence of Falls

Of the 4378 participants with baseline MNA-T2 scores, 881 (20.1%) did not participated in the 2003 survey or did not provide information on falls. Of the remaining 3497 participants, 659 reported having at least one fall in the one-year period before the interview, yielding a cumulative incidence of 18.8%, and participants who were not well-nourished had a higher risk (31.8% vs. 17.9%, p<0.001).

### Risk Factors for Falls

Through uni-variate logistic regression analyses, we found “not well-nourished” (MNA-T2 score ≤23.5), female gender, older age, no spouse (unmarried, widowed, divorced, or separated), less than 10 years of formal education (no high school education), smoking, regular alcohol consumption, hypnotics use, assistive devices use, CES-D score >9, history of falls, hospital stays during past 12 months, ED visits during past 12 months, difficulties in ADL, and difficulties in IADL were potential risk factors for falls (Table 5).

**Table 5:** Univariate analyses of potential risk factors for falls.

Factors	Odds Ratio	95% Confidence Interval	P Value
Not well-nourished (MNA-T2^a^≤23.5)	2.55	1.90, 3.43	<0.001
Female gender	1.79	1.50, 2.13	<0.001
Age (year)	1.04	1.03, 1.05	<0.001
No spouse^b^	1.61	1.34, 1.94	<0.001
Years of formal education			
≤6	2.54	1.55, 4.15	<0.001
7–9	2.05	1.17, 3.59	0.012
10–12	1.56	0.86, 2.83	0.144
>12	1.00	Reference	
Living alone	1.20	0.87, 1.66	0.258
Smoking	0.72	0.58, 0.89	0.002
Regular alcohol consumption	0.72	0.58, 0.88	0.001
No routine exercise	1.13	0,95, 1.34	0.168
Neuroactive medications use	1.17	0.64, 2.11	0.613
Hypnotics use	1.73	1.23, 2.42	0.001
Sedative use	1.47	1.01, 2.15	0.047
Calcium supplement consumption	1.22	0.98, 1.52	0.081
Assistive devices use	2.95	2.19, 3.98	<0.001
CES-D^c^ >9	2.11	1.71, 2.60	<0.001
History of falls	2.25	1.81, 2.79	<0.001
Hospital stays during past 12 months	1.80	1.44, 2.24	<0.001
ED visits^d^ during past 12 months	1.57	1.21, 2.04	0.001
Difficulties in ADL^e^	3.24	2.29, 4.58	<0.001
Difficulties in IADL^f^	2.61	2.18, 3.12	<0.001

a Score for the Mini Nutritional Assessment Taiwan Version 2.

b Including those who were unmarried, widowed, divorced, or separated.

c Geriatric Depression Score measured by the short version of the Center for Epidemiologic Study Depression Scale (CES-D).

d Visits to the emergency departments of hospitals.

e Activities of daily living.

f Instrumental activities of daily living.

Through multi-variate logistic regression analyses, in the full model, the independent risk factors for falls included “not well-nourished,” female gender, older age, years of formal education, history of falls, hospital stay during past 12 months, and difficulties in IADL (Table 6). In the reduced model, the independent risk factors and associated odds ratios (ORs) included 1.73 (95% confidence interval [CI], 1.23, 2.42) for “not well-nourished,” 1.57 (1.30, 1.90) for female gender, 1.03 (1.02, 1.04) for one-year older, 1.55 (1.22, 1.98) for history of falls, 1.34 (1.05, 1.72) for hospital stay during the past 12 months, 1.66 (1.07, 2.58) for difficulties in ADL, and 1.53 (1.23, 1.91) for difficulties in IADL (Table 6).

**Table 6:** Multiple logistic regression analyses of associated factors of falls.

Risk Factors	Full Model		Reduced Model	
	Odds Ratio	95% CI^a^	Odds Ratio	95% CI
Not well-nourished (MNA-T2^b^≤23.5)	1.58*	1.10, 2.26	1.73**	1.23, 2.42
Female gender	1.53**	1.19, 1.96	1.57***	1.30, 1.90
Age (year)	1.03***	1.01, 1.04	1.03***	1.02, 1.04
No spouse^c^	1.04	0.82, 1.32	NA^h^	NA
Years of formal education			NA	NA
≤6	1.73*	1.03, 2.93		
7–9	1.66	0.92, 2.98		
10–12	1.50	0.81, 2.78		
>12	1.00	Reference		
Living alone	1.06	0.73, 1.53	NA	NA
Smoking	1.06	0.81, 1.38	NA	NA
Regular alcohol consumption	1.14	0.89, 1.45	NA	NA
No regular exercise	0.98	0.81, 1.19	NA	NA
Neuroactive medications use	1.15	0.59, 2.22	NA	NA
Hypnotics use	1.24	0.83, 1.83	NA	NA
Sedative use	0.95	0.61, 1.48	NA	NA
Calcium supplement consumption	1.21	0.95, 1.54	NA	NA
Assistive devices use	1.02	0.67, 1.54	NA	NA
CES-D^d^ >9	1.17	0.91, 1.51	NA	NA
History of falls	1.55***	1.21, 1.98	1.55***	1.22, 1.98
Hospital stay during past 12 months	1.39*	1.05, 1.83	1.34*	1.05, 1.72
ED visits^e^ during past 12 months	0.87	0.62, 1.22	NA	NA
Difficulties in ADL^f^	1.62	0.99, 2.64	1.66*	1.07, 2.58
Difficulties in IADL^g^	1.48**	1.17, 1.86	1.53***	1.23, 1.91

a 95% confidence interval.

b Score for the Mini Nutritional Assessment Taiwan Version 2.

c Including those who were unmarried, widowed, divorced, or separated.

d Geriatric Depression score measured by the short version of the Center for Epidemiologic Study Depression Scale (CES-D).

e Visits to the emergency departments of hospitals.

f Activities of daily living.

g Instrumental activities of daily living.

h Not applicable.

*p<0.05; **p<0.01; ***p<0.001.

## Discussion

In this study, using the MNA-T2 score as the indicator, we found that nutritional status was a predictor of falls in Taiwanese 53 years or older living in the community. While studies on this issue are limited, some studies in other countries had also identified malnutrition as a risk factor for falls in community-dwelling older people. A study in Canada found that the level of nutritional risk was a determinant of falls [22], and a study in the Netherlands found that malnutrition was a predictor of falls [2], which was supported by the fact that the malnourished elderly with nutritional intervention had a lower risk of falls than those without intervention [25].

MNA-T2 adopts population-specific anthropometric cut-offs and can effectively predict the nutritional status of older Taiwanese people [39], [40], [43], [45]. In comparison with individual anthropometric measurements, the MNA-T2 score, which includes MAC and CC along with other indicators, provides a more comprehensive measurement of the nutritional status. For example, waist circumference is regarded as a nutritional indicator [42], and a larger waist circumference has been identified as a risk factor for falls [5]. However, people with malnutrition may have smaller, instead of larger, waist circumference [46]. Likewise, in comparison with individual biochemical indicators such as the serum albumin level [5], [49] or individual physiological parameters such as systolic blood pressure [13], MNA-T2 score can also provide a more comprehensive measurement of the nutritional status.

Previous studies found that malnutrition was able to predict functional decline of the elderly [48], [50]–[52], which has been identified as a risk factor for falls [4], [11]. Many studies have shown that reduced mobility in general is a risk factor for falls [23], [25]. More specifically, a recent systematic review of literature on Chinese living in Taiwan, Hong Kong, Macao, Singapore, and China found that both declines in ADL and IADL were risk factors for falls in older Chinese people [4]. However, after adjusting for difficulties ADL and IADL, our study still observed an association between nutritional status and falls, and this is compatible with the finding in a study in the Netherlands that malnutrition was a risk factor for falls independent of the level of activity [25]. People with malnutrition also tend to have nutrition-related health problems [28], which is another risk factor for falls in the older people [2], [4], [9], [14], [17]. In particular, a study in Australia found that 20.3% of fallers in older people had one or more hospital admissions during past 12 months [53], which is compatible with the finding in our study. However, we still observed an association between nutritional status and falls after adjusting for hospital stay during the past 12 months. This might suggest that some nutrition-related health problems which are not serious enough to lead to hospital stays may still increase the risk of falls in order people. For example, a recent review of literature found that vitamin D supplementation in people with lower vitamin D levels was associated with fewer falls in community-dwelling people with risk factors for falling [54]. In addition, previous studies found that malnutrition was associated with decreased muscle power [27] and impaired balance [55], and both of them were risk factors for falls in the elderly [1], [3], [15], [17], [22].

In addition to nutritional status, we found the female gender was an independent risk factor for falls in older people. A previous study on community-dwelling older people in Taiwan found that the female gender was associated with an OR of 1.94 for falls after adjusting for other risk factors [5], and a study on the elderly community dwellers in Singapore also found that the female gender was a risk factor for falls [11]. In fact, the systematic review of literature on Chinese living in Taiwan, Hong Kong, Macao, Singapore, and China has shown that the female gender was a risk factor for falls in older Chinese people, with ORs ranging from 1.5 to 2.9 [4]. This is also true in some other countries. For example, a study in Finland found that the proportion of women seeking medical treatment due to a fall at least once during an one-year period was higher than that of men (4.4% vs. 2.5%) [10], and a study in Japan also found that the proportion of women having at least one fall during a three-year period was higher than that of men (24.1% vs. 17.4%) [56].

In our study, age was a risk factor for falls, and the OR associated with a one-year increase in age was 1.03 after adjusting for other risk factor in older people. A previous study of community-dwelling older people in Taiwan also found that the OR associated with a one-year increase in age was 1.03 after adjusting for other risk factors [5]. In addition, a population-based study in Hong Kong found that old age was an important independent predictor of falls in the Chinese elderly [3], and the review of literature on Chinese living in Taiwan, Hong Kong, Macao, Singapore, and China found that older age was a risk factor for falls [4]. Similar findings were observed in other populations; for example, a study of community-dwelling elderly adults in the USA found that age was a risk factor for falls in both genders [9].

As in our study, a population-based cohort study in Hong Kong found that history of falls was an important independent predictor of falls in the elderly [3], and the review of literature on Chinese living in Taiwan, Hong Kong, Macao, Singapore, and China found that history of falls in the past 12 to 18 months was a risk factor for falls in older people, with relative risks (RRs) ranging from 1.7 to 13.1 [4]. Studies in the USA also found that a history of fall was a significant predictor of being a faller [20], [21]. In particular, a systematic review in the USA found that patients who had fallen in the past year were more likely to fall again, which supports the findings in our study [1].

Some studies found exercise programs can reduce the risk of falls [11], [13], but effective exercise programs included exercises that challenged balance and used a high amount of exercise [10], [57]. Unplanned exercise might not be as effective as those programs for community-dwelling older people and might even increase the chance of falls due to the exercise itself. Therefore, our finding that regular exercise had an OR of 0.98 (near the null value 1.00) after adjusting for other potential risk factors in the full model is reasonable.

The current study found neuroactive medication use did not predict falls, which was similar to the finding in a previous study [58]. In the full model, we found a slight increase of risk associated with neuroactive medications, and the adjusted OR (1.15) was very close to the crude OR (1.17). Therefore, we believe the effect of neuroactive medications, if existed, was too small in this study population to reach a statistical significance.

The cumulative incidence rate of falls during the one-year period in our study was similar to those in previous studies in Taiwan [5], Hong Kong [3], and Singapore [11]. In fact, a systemic review of studies on Chinese living in Taiwan, Hong Kong, Macao, Singapore, and China found that the one-year cumulative incidence ranged from 14.7% to 34% with a median 18% [4], which is similar to our finding of 18.8%.

This study is one of the limited longitudinal studies of falls on a sample representing the whole nation, and the number of risk factors studied was more than those in most of the previous studies, which makes the results more reliable. Still, our study has limitations. In particular, we used self-reported data, and the incidence of falls might be underestimated because some older people might not recall all the falls. Likewise, the assessment of nutritional status included some self-reported data, and there might be potential biases in spite of the fact that some validation studies on MNA-T2 had been conducted [38]–[40], [45]. Although MNA-T2 has been shown to have high validity in predicting the nutritional status of older Taiwanese people, it could not effectively identify obesity, because the highest cut offs are 30 cm in men and 27 cm in women for CC and 23.5 cm in men and 22.0 cm in women for MAC; neither of them can be used for identifying obesity. Therefore, we were unable to evaluate and adjust for the effects of obesity. As a result of this categorization, the obese participants were categorized as “well-nourished” in our final model, and this might lead to underestimation of the effects of “not well-nourished” because obesity has been identified as a risk factor for falls. Since we identified “not well-nourished” as a risk factor for falls even when its associated risk might be underestimated, this limitation in categorization did not affect our conclusion. Whereas the participants who had MNA-T2 scores were younger, we studied the occurrence of falls after the measurement of MNA-T2, and therefore the younger age of the participants would not introduce selection bias in our conclusion. In addition, since the final model included age, the younger age of the participants would not introduce confounding bias in our conclusion. The participants who were included in the follow up had higher proportions of males, age between 53 and 74 years old, and having a spouse. Likewise, we studied the occurrence of falls during the follow-up, and therefore the differences would not introduce selection bias to our conclusion. In addition, since the full model included gender, age, and no spouse but still found a significant effect of nutritional status, these differences would not introduce confounding bias to our conclusion. On the other hand, data on many of potential confounding factors were collected 4 years before the assessment of falls, and some of them might have changed over this time period and lead to misclassification. Even though we have evaluated and adjusted for many potential risk factors for falls in the elderly, we did not have data on some other risk factors such as visual impairment [5], [11], [12], [19]. Therefore, further studies that can adjust for those factors should be conducted in the future.

In conclusion, we found that nutritional status was a predictor of falls in older people living in the community, and its effects were independent of other risk factors, including female gender, older age, history of falls, hospital stay during past 12 months, difficulties in ADL, and difficulties in IADL. Further studies are warranted to identify the types of nutritional interventions that can help prevent falls in the elderly.
